# Using Games to Simulate Medication Adherence and Nonadherence: Laboratory Experiment in Gamified Behavioral Simulation

**DOI:** 10.2196/47141

**Published:** 2024-09-24

**Authors:** Umar Taj, Aikaterini Grimani, Daniel Read, Ivo Vlaev

**Affiliations:** 1 Behavioural Science Group, Warwick Business School, University of Warwick, Scarman Road, Coventry, CV4 7AL, United Kingdom, 44 024-765-24498

**Keywords:** behavior change, experimental modeling, gamification, medication adherence, antibiotics, games, medication, testing behavior, clinical outcome, simulate, diagnosis, devices, symptoms, tool

## Abstract

**Background:**

Medical nonadherence is a significant problem associated with worse clinical outcomes, higher downstream rehospitalization rates, and a higher use of resources. To improve medication adherence, it is vital for researchers and practitioners to have a solid theoretical understanding of what interventions are likely to work. To achieve this understanding, we propose that researchers should focus on creating small-scale laboratory analogs to the larger real-world setting and determine what interventions, such as nudges or incentives, work to change behavior in the laboratory. To do this, we took inspiration from the literature on serious games and gamification and experimental economics. We call our approach “gamified behavioral simulation.” In this paper, we modeled everyday life as the state of being engaged in a simple but addictive game, illness as being interruptions to the functionality of that game, treatment as being a series of actions that can be taken to prevent or mitigate those interruptions, and adherence as sticking to a prescribed rule for the application of those actions.

**Objective:**

This study carries out a behavioral diagnosis of the medication adherence problem through a theoretically informed framework and then develops the gamified behavioral modeling approach to simulate medication nonadherence.

**Methods:**

A laboratory experiment was conducted using a modified popular and addictive open-source video game called “2048,” which created an abstract model for the medication adherence behavior observed in real life. In total, 509 participants were assigned to the control and 4 intervention groups (“incentive” group, “reminder” group, “commitment device” group, and “elongated duration for symptoms” group).

**Results:**

The results of the modeling experiment showed that having theoretically informed interventions can increase the likelihood for them to be successful. In particular, there is evidence that the use of reminders improves the medication adherence rates for patients, and the same result was found in the modeling experiment, as they improved adherence significantly by 23% (95% CI −33.97% to −11.72%; *P*<.001). However, providing an incentive did not improve the adherence rate. We also tested the use of commitment devices, which, in line with real-world evidence, did not improve adherence rates. The fourth treatment tested elongated duration for symptoms, which attempted to show the power of modeling experiments where we test a what-if scenario that is extremely difficult to test in a real setting. The results indicated that if symptoms last longer, people did not adhere more to their medication regimen.

**Conclusions:**

Gamified behavioral simulation is a useful tool to explain real health behaviors and help in identifying which interventions are most likely to work in a randomized trial.

## Introduction

### Overview

Medication adherence is the extent to which the patient’s behavior matches the agreed recommendations from the prescriber. There is consistent evidence that regardless of what is being treated, nonadherence is associated with worse clinical outcomes and higher downstream rehospitalization rates [1]. Yet, several reviews have found that medication adherence among patients is low, especially in low-income countries [1-3]. In the United States, for example, 51% of patients with hypertension adhere to their medication, but the corresponding numbers in Gambia, Seychelles, and China are 27%, 26%, and 43%, respectively [4-8]. Even in the developed world, medication nonadherence is expensive. The annual cost to the UK government has been estimated at £150 million (approximately US $270 million in 2005); for the United States, this figure is over US $1 billion [3].

The cause of medication nonadherence varies among patients and can be categorized as intentional or unintentional [9]. Intentional nonadherence involves patients who decide consciously not to take medication as instructed, based on perceptions, feelings, or beliefs [10]. Unintentional nonadherence occurs when the patient wants to take their medication as instructed but fails to do so. The most common factors associated with nonadherence include forgetfulness (50%), having other medications to take (20%), and being symptom-free (20%) [11]. To the extent that nonadherence is unintentional, patients are likely to welcome any nudge that helps them adhere to their doctor’s recommendations.

New and innovative strategies are essential to successfully improve patient adherence to treatment. One potentially effective strategy for understanding patient medication adherence is the use of serious games and gamification [12] or the introduction of gaming elements into the medication experience. Games motivate users into engaging in an activity with a higher intensity and duration [13]. Game elements are activities, behaviors, and mechanisms designers incorporate into a specific context to create a gameful experience [13]. Introducing gaming elements into a nongaming context has the potential to transform routine tasks into more enjoyable and motivating experiences [14]. The aim of this study is to conduct a behavioral diagnosis of medication adherence issues using a theoretically informed framework and then to design, implement, and assess a game-based setting for simulating nonadherence behavior.

A range of studies have explored the use of games and technology (mainly via mobile health apps) to understand and improve treatment adherence. Tran et al [15] found that these features can lead to improved or sustained medication adherence but noted significant heterogeneity in patient populations and methodologies. de Vette et al [16] highlighted the potential of games to engage older people in telemedicine, while Brown et al [17] called for further research to understand the impact of game features on program adherence in web-based interventions for mental health. These studies collectively underscore the potential of games and incentives in improving medication adherence.

This paper takes inspiration from the literature on serious games and gamification and experimental economics. We call our approach “gamified behavioral simulation.” We model everyday life as the state of being engaged in a simple but addictive game, illness as being interruptions to the functionality of that game, treatment as being a series of actions that can be taken to prevent or mitigate those interruptions, and adherence as sticking to a prescribed rule for the application of those actions. As far as we are aware, no previous research has addressed whether serious games can be used to model (simulate) medication adherence behavior and test the effectiveness of interventions to improve such adherence. This is a crucial gap in the literature that our study addresses.

### Background

Existing literature indicates that interventions to improve medication adherence have had mixed results [3,8]. The Medical Research Council proposes that the development of a behavior change intervention should follow the same cycle as drug development: (1) a theory behind the design of behavioral intervention; (2) followed by modeling of the problem or behavior; and (3) finally, a randomized controlled trial (RCT) and implementation of the intervention [18].

Interventions developed to bring about behavior change often offer limited practical value, as they lack a theoretical basis for their selection and development [19]. The Medical Research Council proposes that an important early task for a researcher is to develop a theoretical understanding of the underlying process and constructs that might bring about behavior change. This helps in clearly understanding how successful interventions have had their effect, that is, which behavior change processes can be attributed to the observed change. A theoretical underpinning further allows the researcher to argue for the selection of a particular intervention [18].

The Medical Research Council proposed that this “theory” stage should be followed by a “modeling” stage, which can be considered as the equivalent to “testing with mice” in the drug development cycle. Modeling allows the researcher to investigate and identify the exact mechanisms that are bringing about the behavior change. It makes possible the study of isolated effects of different interventions. Modeling thus allows the researcher to make complex phenomena manageable and create knowledge about the underlying mechanisms of behavior change that might be quite difficult to uncover otherwise [18]. It is only once a clear understanding of what works has been achieved through the modeling stage that an RCT should be carried out to empirically test the intervention.

In this study, we focus on the “modeling” stage for developing behavior change interventions to increase patient’s adherence to antibiotic medication. It is common in behavioral science and economics to model real-life behaviors in a laboratory setting in an attempt to control and make tractable the phenomenon that the researcher is interested in investigating. However, introducing this modeling approach in the development of complex interventions is not commonly found in the health behavior change literature [19]. We argue that using gamified behavioral simulation to first model treatment behavior can provide important information about the choice and design of the behavior change intervention to be tested in an RCT in a short time with a low cost.

Our experimental model for the nonadherent behavior in patients taking antibiotics was inspired by the conceptual approach developed by Kessler and Roth [20]. They used an abstract experimental interaction to model the effectiveness of the priority rule in increasing the registration of organ donors. An organ allocation policy that prioritizes registered donors on waiting lists was found to significantly boost donor registration. Their setting did not involve actual organ donation decisions and neither did they use any organ donation terminology during the experiment. However, they imposed real (monetary) costs to correspond to the analogous costs associated with decisions to donate or receive an organ. Their results showed that the priority rule condition had a significant positive impact on their laboratory-based “organ donation–like” decisions, and they used this to make a strong case in their paper to introduce the priority rule in the organ donation policy present in the United States. Subsequent work in the field indeed suggested that this laboratory method, designed without making any direct reference to organ donation, predicted organ donation attitudes and preferences [21] and even became the basis for policy decisions in at least 1 country [22].

We adopted a similar approach. The aim of this study was to develop an abstract experimental task that can capture the key elements of a real-world setting and thus simulate the nonadherence behavior of the patients taking antibiotic medication. At the heart of the task was a “game,” which enabled us to make the experiment engaging and to incentivize behavior. This simulation served as a platform to test multiple interventions to positively increase the target behavior.

Our research question addresses a significant challenge in gamification technology: whether gamified behavioral modeling is a useful tool to explain real health behaviors and help in identifying which interventions are most likely to work in the real-world [23,24]. This challenge stems from the need to validate that behaviors observed within a gamified environment accurately reflect those in real-world settings. Additionally, it ensures that the motivational elements used in serious games are effective in driving the desired health behaviors and can be predictive of the outcomes of interventions in randomized trials. Addressing this challenge is crucial for the advancement of gamified applications in health-related fields, where the ultimate goal is to positively influence health outcomes and behaviors.

To address this question, we examined a range of interventions—those that have yielded both successful and unsuccessful outcomes in real-world trials—and assessed whether our gamified behavioral simulation can mirror these varied patterns of results. Therefore, our hypothesis was that our gamified behavioral simulation can accurately replicate these results, which implies that it would be a useful tool for predicting the success of interventions in the field.

Note that this approach differs from traditional methods to develop and test medication adherence interventions, which usually involve understanding barriers to action (adherence) and designing field trials to test interventions that address those behavior change challenges [25-27]. These interventions include patient education, medication regimen management, clinical pharmacist consultation, cognitive behavioral therapies, incentives, and various technology-based intervention and measurement components such as medication-taking reminders, support messages, and adherence measurement methods (eg, electronic drug monitors [pill bottles], sensor systems, and proximity sensing) [26]. Such field trials are costly and often cannot test every possible intervention strategy. Thus, evaluating our gaming methodology is essential, as it allows us to explore certain hypotheses about medication adherence within a controlled laboratory environment, where actual medication decisions are not involved. While not all aspects of medication adherence can be abstracted for study, there are critical elements of the adherence decision process that cannot be consistently manipulated in real-world settings, yet are amenable to manipulation in a laboratory setting (such as, different incentive schemes or elongated duration for symptoms).

## Methods

### Experiment Design

The laboratory experiments were conducted in December 2015 by Gallup Pakistan at 2 laboratories in Islamabad and Karachi. The laboratory experiments involved using a (then) popular and addictive open-source video game called “2048.” The game’s aim is to move numbered tiles in such a way that the total adds up to 2048 (Figure S1 in Multimedia Appendix 1).

The original 2048 game was modified to create an abstract model for the medication adherence behavior observed in real life. The game is the analog of “everyday life.”

### Participant Recruitment

The sample (N=509; n=305, 60% male and n=204, 40% female) was recruited through Gallup Pakistan. They were screened on the basis of whether they were able to browse on the internet, as the laboratory experiments requested to play a computer game (each of the control and treatment groups had the same representative proportions). Gallup Pakistan provided transport facilities to any participants who requested it. This was especially the case with female participants, as it was quite difficult to recruit them otherwise. If necessary, the timing of running the experiment was adjusted to accommodate participants after office hours. All participants provided written informed consent.

### Procedure

The participants were asked to open the original 2048 game tab and practice for 10 minutes. At the end of the practice round, each participant was asked to close the original 2048 game tab and was directed to open an instruction video tab. The instructional video in Urdu (the national language of Pakistan) was made following a number of pilot tests (Multimedia Appendix 1) and extensive feedback from participants. It was recorded using CamStudio (Rendersoft Development). The instructional video explained in detail how to begin playing the experimental game and gave a demo of the experimental game. Three videos were made. The video for the control group was also used for the “commitment device” treatment condition and the “elongated duration for symptoms” treatment condition. The content of the “incentive” and “reminder” videos were the same except for some condition-specific information. Once the participants were finished watching the instructional video, they were asked to begin the experimental game.

The total duration of the game part of the experiment was 14 minutes and 30 seconds, and the participants were instructed to enter the code every minute or 14 times in total. The 14 code entries simulated a typical 7-day antibiotic medication course where patients are prescribed to take the pill twice a day with roughly 12-hour intervals. However, the inclusion of the initial practice rounds, the instruction video, and the payment process brought the whole task to 30 minutes on average.

### Control Condition

When participants started the game, after the instructional video, the screen was blurred, making it very difficult for them to play the game. This blurriness simulated the onset of illness. To simulate the use of medication, participants were provided with a code, which they were required to enter every minute to clear the screen. The screen became clearer each time the code was entered. However, halfway through the game, the screen remained clear even though the participants were still expected to enter the code. If the code was not entered, however, there was a chance of “relapse,” and the screen became blurry again.

As an analog to the pill pack, a pill counter was displayed on the left of the screen, showing how many times the code had been entered and how many times remained. Participants received a total of 14 opportunities to enter the code correctly, 1 every minute. If participants failed to adhere to this regimen, then, after the 14 opportunities, their codes become ineffective. This corresponded to the cost of forgetting to take one’s medication. On the top left corner of the screen, a timer was displayed with the time elapsed since the start of the game. The timer was there to assist the participants in keeping track of time.

The show-up fee was Rs 80 (£0.50, approximately US $0.77 in 2015), and it was already included in the earnings; participants started the game with a score of 0 and earnings of Rs 80 (£0.50, approximately US $0.77 in 2015). Participants were rewarded on how well they scored in the 2048 game. The maximum money that participants could earn was Rs 500 (approximately £3 or US $4.59 in 2015). The final score and earning of the participant showed up on the screen along with their participant number, which served as an ID.

Participants were allowed to play the game as many times as possible during the duration of the experiment (which was 14 minutes 30 seconds). Once a player had no moves left on the board, a message box popped up on the screen giving the participant an option to restart the game (Multimedia Appendix 1).

To examine whether gamified behavioral modeling is a useful tool to explain real health behaviors and predict which interventions are most likely to work, we examined a range of interventions that have yielded both successful and unsuccessful outcomes in real-world trials, aiming to improve antibiotic medication adherence.

### Treatment Condition 1: Incentive

For the “incentive” treatment condition, the design and mechanics of the experiment were exactly the same as the control experiment except that participants were given an incentive of Rs 5 (£0.02, approximately US $0.03 in 2015) every time they entered the correct code on time. This was an extra bonus on top of their usual earnings in the game, and the increment would show up in the earnings box on the top left corner of the screen. In addition, when participants entered the code correctly and on time, a message would flash on the screen informing them that they earned a bonus of Rs 5 (£0.02, approximately US $0.03 in 2015).

The instructional video before the “incentive” version of the game explicitly mentioned the Rs 5 (£0.02, approximately US $0.03 in 2015) bonus that they would earn upon entering the code correctly on time. The video also showed one such instance where the code was entered correctly on time and the earnings increased by Rs 5 (£0.02, approximately US $0.03 in 2015).

### Treatment Condition 2: Reminder

For the “reminder” treatment condition, the design and mechanics of the experiment were exactly the same as the control experiment except that a message box would pop up on the screen when it was time to enter the code. The message box informed the participant that it was time to enter the code. This message box would appear in the middle of the board and stay for a few seconds before disappearing.

The instructional video for the “reminder” version of the game explicitly mentioned that a reminder message would show up when it would be time to enter the code. The video also showed one such instance where it was time to enter the code and the message box popped up on the screen.

### Treatment Condition 3: Commitment Device

For the “commitment device” treatment condition, the design and mechanics of the experiment were identical to the control experiment except that at the start of the experiment, participants were asked to sign a sticker stating that they committed to entering the code as prescribed. The sticker was then pasted on the laptop they were using to play the experimental game. Participants watched the same instruction video as in the “control” condition.

### Treatment Condition 4: Elongated Duration for Symptoms

An additional treatment condition was introduced in which symptoms took twice as long to disappear compared to the control condition. For the “elongated duration for symptoms” treatment condition, the rest of the design and mechanics of the experiment were exactly the same as the control experiment. Participants watched the same instruction video as in the “control” condition.

### Ethical Considerations

The study was approved by the University of Warwick’s ethics committee (ethical application reference 100/15‐16). All procedures were conducted in accordance with the relevant guidelines and regulations. Strict ethical and legal standards were upheld, ensuring that all personal information was securely stored, treated confidentially, and anonymized. Informed consent was obtained from all participants, permitting the use and publication of their data in this research. Participants received a show-up fee of Rs 80 (approximately US $0.77 in 2015) and had the opportunity to earn up to Rs 500 (approximately £3 or US $4.59 in 2015) based on their performance in the game. Participation in the study was entirely voluntary, and participants retained the right to withdraw at any time without any obligation for further contact from the study staff after withdrawal.

## Results

**Figure 1.**
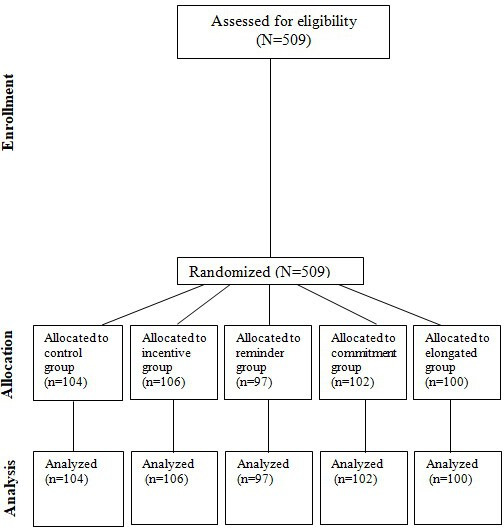
CONSORT (Consolidated Standards of Reporting Trials) diagram.

We report results from 509 participants who participated in our laboratory experiment (Figure 1). There were 104 participants in the control group, 106 in the “incentive” group, 97 in the “reminder” group, 102 in the “commitment device” group, and 100 in the “elongated duration for symptoms” group.

Planned comparisons (ANOVA) were carried out to determine significant changes in adherence rates between the control group and treatment groups using the Bonferroni corrections post hoc test. The results showed that the adherence rate differed among the conditions (*F*_4,504_=10.63; *P*<.001; *n*^2^_p_=0.078). The adherence rate in the control group was 44% (mean 44.16%, SD 27.45%), and similar adherence rates were seen in the “incentive” group (mean 52.02%, SD 29.41%), the “commitment device” group (mean 45.17%, SD 26.50%), and the “elongated duration for symptoms” group (mean 53.57%, SD 29.05%). In particular, the commitment device did not bring any change in the adherence rate compared to the control group (1%, 95% CI −11.99% to 9.98%). In the “incentive” as well as in the “elongated duration for symptoms” treatment group, the adherence rate improved by 8% (95% CI −18.74% to 3.02%) and 10% (95% CI −20.45% to 1.63%), respectively; however, the results were insignificant (*P*=.42 and *P*=.17, respectively). On the contrary, reminders had a different adherence rate (mean 67.01%, SD 27.17%) compared to the control group, as they improved adherence significantly by 23% (95% CI −33.97% to −11.72%; *P*<.001). The finding here suggests that simply reminding people to take their antibiotic medication can improve medication adherence significantly (Table 1).

Regarding the code entries, 63% (n=4489) were correct and on time across all conditions, while 21% (n=1497), although correct, were entered at the wrong time. This seems like a fairly reasonable result, as it was expected that some patients might take their pill at the wrong time. Only 7% (n=499) of the code entries consisted of wrong codes, which translated to a few patients taking the wrong pill. This result can be justified as patients who are on complex medication regimens (such as, for tuberculosis) do sometimes take the wrong pill.

The experiment included a consequence for nonadherence in the form of the screen relapsing to being blurry. To recall, once the participants’ screen was cleared, if they failed to enter the code, there was a 2% chance of “relapse” or the screen becoming blurry by 25%. The probability of “relapse” doubled each time the code was not entered. There were 73 participants in total who experienced relapse (each of these participants only experienced relapse once).

Our expectation was that once a participant had experienced relapse, they would be more adherent, but it seems that except for the incentive condition, adherence rates dropped after relapse. However, there may be an explanation for this result. Table 2 shows the round when relapse occurred for different participants. It can be seen that for more than half of the participants, relapse occurred when they only had 1, 2, or 3 code entries left. Since they knew that the game would end soon, they might have decided to forego entering the code and instead focus on the 2048 game. Since most participants experienced relapse very close to the end of the experiment, drawing meaningful insight into the relationship between relapse and adherence rate might not be appropriate.

**Table 1.** Adherence rate of the control group and the intervention groups.

Group	Sample size, n (%)	Adherence rate (%)
Control group	104 (20)	44
Incentive group	106 (21)	52
Reminder group	97 (19)	67
Commitment device group	102 (20)	45
Elongated duration for symptoms group	100 (20)	54

**Table 2.** Adherence rates across the 5 conditions in the experiment.

Round when relapsed	Adherence rates (%)
7	4.1
8	5.5
9	4.1
10	19.2
11	8.2
12	16.4
13	21.9
14	20.5

## Discussion

### Overview

We intended to carry out a behavioral diagnosis of the medication adherence problem through a theoretically informed framework and then develop a model to simulate the nonadherence behavior. This constitutes the second stage of the theory-modeling-RCT cycle for developing behavior change interventions. The results of the modeling experiment showed that having theoretically informed interventions increased the likelihood for them to be successful. Furthermore, modeling can also help in identifying which interventions are most likely to work in the RCT.

It is worth discussing how good the 2048 experiment has been in modeling the nonadherence behavior. The modeling experiment seemed to have generated the same patterns that are expressed by patients such as forgetting to take the pill or adhering less to the regimen once the symptoms disappear. The results of the modeling experiment reflect this behavior of patients quite closely among the participants as well. The adherence rate found among the control group of the game experiment was 44%, which is quite similar to the adherence rate found among patients. While this statistic alone does not fully confirm the effectiveness of the gamification model, it is important to note that if the control group’s adherence rate had been either very low or very high, it would have indicated that the 2048 experiment was not accurately modeling nonadherence behavior. Among the treatment conditions that were tested, it seems that reminders are only effective in increasing adherence rates.

### Comparison to Prior Work

The results of the modeling experiment showed that providing incentives did not improve the adherence rate. Providing incentives was chosen as one of the treatment conditions because there is a lack of reinforcement for patients to continue adhering to their antibiotic medication once the symptoms disappeared [28]. However, Klein [29] maintains that rewarding behavior with money may have the undesirable effect of devaluing the intrinsic benefits of adherence, creating an even higher barrier to long-term adherence. Ideally, incentives should provide frequent, small (but tangible) rewards [30-32].

The results of the modeling experiment showed that providing reminders improves the adherence rate. There is evidence that the use of reminders improves the medication adherence rates for patients [33,34] because forgetting is the most prominent reason for nonadherence [28]. Using SMS text messaging for medication reminders appears to have a significant positive effect on medication adherence in other clinical areas such as, for example, mental health [35,36], with enduring effects [34]. The effectiveness of interventions using electronic reminders to improve adherence to antibiotics has not been tested in the field for real, so our experiment provides a promising indication that such an intervention is very likely to be effective.

While the use of reminders increased the adherence rate in the modeling experiments, we wanted to turn the tables and model an intervention that was yet to be carried out in a real-world setting. This motivated our selection of the use of commitment devices as one of the treatment groups in the modeling experiment. Commitment devices have been used in various contexts, such as smoking cessation, weight loss, exercise, and savings. Some studies have also explored the use of commitment devices for medication adherence among patients with chronic conditions, such as HIV, diabetes, or hypertension [28]. The results have been mixed, but some evidence suggests that commitment devices can improve medication adherence and health outcomes, especially when combined with other strategies, such as reminders, education, and incentives. Therefore, we aimed to test this strategy in our setting. We used the same protocol that was followed by the Department of Health and Boots UK in their RCT, and the results showed that the commitment sticker did not improve the adherence rates of the participants in the modeling experiment. Interestingly, a few months later, when the results of the RCT came out, it was found that the commitment sticker did not bring about any improvement in the adherence rates among patients as well. From the results obtained in the modeling experiment and their comparison with real-world analogs, it seems that the modeling experiment was able to simulate the nonadherence behavior of the patients.

The last treatment group of “elongated duration for symptoms” really shows the power of modeling experiments where we test a what-if scenario, which is extremely difficult to test in an RCT setting. The results from the experiment provide a proof of concept that if symptoms last longer people adhere more to their medication regimen. An interesting idea that comes to mind is that maybe the pills can be made in such a way that they keep the patients feeling sick until the last day of their treatment. Interestingly, a behavioral consultancy in India is testing a similar intervention. They are working on the issue of nonadherence to tuberculosis medication (which is an antibiotic medication but for a 6-month period) and have come across the same issue that patients stop taking their pills once the symptoms disappear. Rather than making people keep feeling sick (which is an extreme interpretation of the modeling experiment finding), they introduced an intervention to make patients realize that they are still sick even though the symptoms are gone.

In this study, we did not aim to identify which intervention or combination of interventions can be most effective in increasing medication adherence but rather to develop a model that can provide a platform to test various interventions and select the most effective ones to be included in an RCT. It is quite possible that an intervention is successful in an RCT but not acceptable by the target group. We would suggest that once we know from the modeling experiment which interventions show promise, we should carry out a reality check with the target group and understand how accepting they would be if that intervention was rolled out. In a recent study, researchers conducted an RCT to assess the value of SMS text message reminders as a means to improve medication adherence in patients receiving treatment for the prevention of cardiovascular disease [37]. They found that reminders improved adherence rate by 16%, but the interesting point to note is that for this study they contacted 7004 patients and only 303 agreed to receive text reminders. Although the study showed that reminders significantly improved adherence rates of the patients, the participant numbers also hint that many patients might not be interested in this service if it were to be rolled out. Hence, it is important to think about the acceptability of an intervention at the time of selecting interventions that are carried forward from the modeling stage to the RCT stage.

### Limitations

The participants were screened on the basis of whether they were able to browse on the internet, as the laboratory experiments involved playing a computer game, which could be considered as a limitation. A significant proportion of the Pakistani population does not know how to use a computer, which restricted us to recruiting a representative sample of the computer-literate Pakistani population rather than a representative sample of the whole Pakistani population. Additionally, one argument that can be made against this modeling experiment is that participants might have adopted a strategy, whereby they enter the code to clear the screen, and once the screen is cleared, they cease to enter the code and maximize the time being spent to play the game, thereby maximize their earning in the experiment. However, this cannot be imagined to be a dominant strategy in the real-world setting, as patients do pay heed to the advice given by their doctors and have a high degree of trust and confidence in their doctors.

### Conclusions

For this study, we set up the first behavioral science laboratory in Pakistan. We were keen on having the general public as our participants, and we knew that none of them would ever have participated in an experiment before, making them more receptive to the task. Using a game in the experiment proved to be a very important factor in attracting participants and greatly improved their engagement. As we said earlier, many participants requested to play the game again because they enjoyed it. We believe that more attention should be paid by researchers on how to keep the participants engaged when designing an experiment.

Our investigation answered our research question: whether gamified behavioral modeling is a useful tool to explain real health behaviors and help in identifying which interventions are most likely to work in a randomized trial. We examined a range of interventions that have been tested in real-world settings, and we discovered that our gamified behavioral simulation can model these varied patterns of results. We confirmed our hypothesis that the gamified behavioral simulation can replicate such results, which implies it would be a useful tool for predicting the success of interventions in the field.

Developing behavior change interventions is a complex process and thus requires a systematic way to approach the problem. The process should start from developing a theoretical understanding of the behavior at hand, followed by modeling the behavior to identify the exact mechanisms that might bring about the desired behavior change. Once the most suitable interventions are identified in the modeling stage, then an RCT should be carried out to test the intervention in a real-world setting. In this study, we have showed how this process can be followed in relation to the problem of nonadherence to antibiotic medication. Care must always be taken in extrapolating results from the laboratory to the real world, and caution is particularly called for when the laboratory model abstracts away from some important features such as the feelings of the patients or the environment in which patients are making their decisions. However, generating a hypothesis through simple modeling experiments can help us in developing the most effective interventions that can then be tested in the real-world setting (through RCTs).

## Supplementary material

10.2196/47141 Multimedia Appendix 1 Information detailing the design and methodology.

## Abbreviations

RCT: randomized controlled trial
