# Supplementary material for: Using Games to Simulate Medication Adherence and Nonadherence: Laboratory Experiment in Gamified Behavioral Simulation
Source: JMIR Serious Games. 2024 Sep 24;12:e47141. doi: 10.2196/47141 (PMC11444231; doi:10.2196/47141)
Supplement: Multimedia Appendix 1 [file games-v12-e47141-s001.docx]

**Figure S1.** Screenshot of Original 2048 game.

| 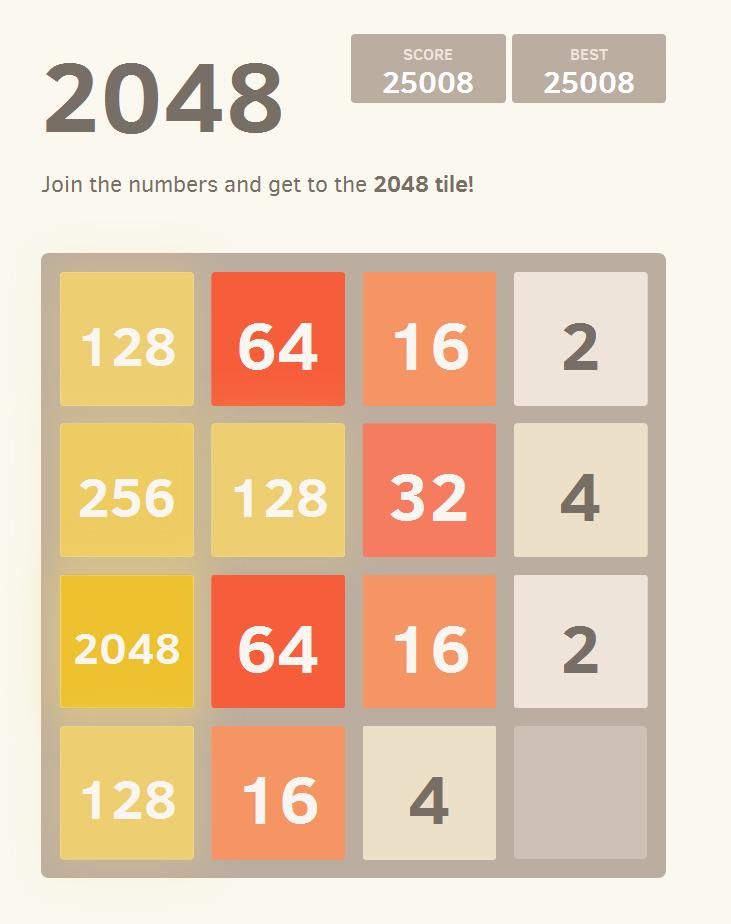 |
| --- |
|  |

**Game “2048 details”**: The game has a 4X4 grid, with numbered tiles that can be moved in four directions (up, down, right and left) by using the four arrow keys on the keyboard. Upon every turn, a new tile appears on one of the empty spots on the board with a value of either 2 or 4. Tiles move as far as possible in the direction of the arrow key that has been pressed until they are stopped by another tile or the edge of the board. If two tiles of the same number collide, then they merge together to form a single tile with the number being the sum of the numbers on the preceding two tiles. So, for example, to reach the final score of 2048, two tiles both carrying the number 1024 has to be merged; and in order to get a tile numbered 1024, two tiles both carrying the number 512 have to be merged and so on. When the player has no moves left, that is, there are no empty spaces left on the board and there are no adjacent tiles with the same number, the game ends. As soon as the game ends, an option appears on the screen for the participant to restart the game.

A scoreboard keeps track of the participant’s score. The participant’s score starts with a score of zero and it increments whenever two tiles with same numbers are merged.

The 2048 game was chosen for our experimental game modelling due to the following reasons:

1. It is an open source game, hence allowing us to modify the game to suit the needs of the experiment.
2. The game is quite intuitive and it only takes a few minutes for a new player to learn how to play.
3. The game is highly engaging, in fact during the course of the actual experiments several participants enquired and requested if they can take part in the experiment again as they really enjoyed themselves.

##### **Control Condition**

When participants start the game, the screen is blurred making it very difficult for them to play the game. This blurriness simulated the onset of illness. To simulate the use of medication, they were given a code which they entered every minute to clear the screen. The screen became clearer each time the code was entered. However, halfway through, the screen became clear while the participants were still expected to enter the code. If the code was not entered, they could “relapse” and the screen became blurry again.

*Blurriness*

When participants start the game, the blurriness was set at 100% and each time they entered the correct code blurriness reduced by 25%. If they fail to enter the code, there was a 2% chance of “relapse”, where the screen became blurry by 25%. The probability of “relapse” doubled each time the code was not entered. This mechanism of “relapse” activates in the experiment after the screen becomes clear for the first time. If a “relapse” had occurred, the participants were able to clear the screen again by subsequently entering a correct and on time code (see Figure S2).

| 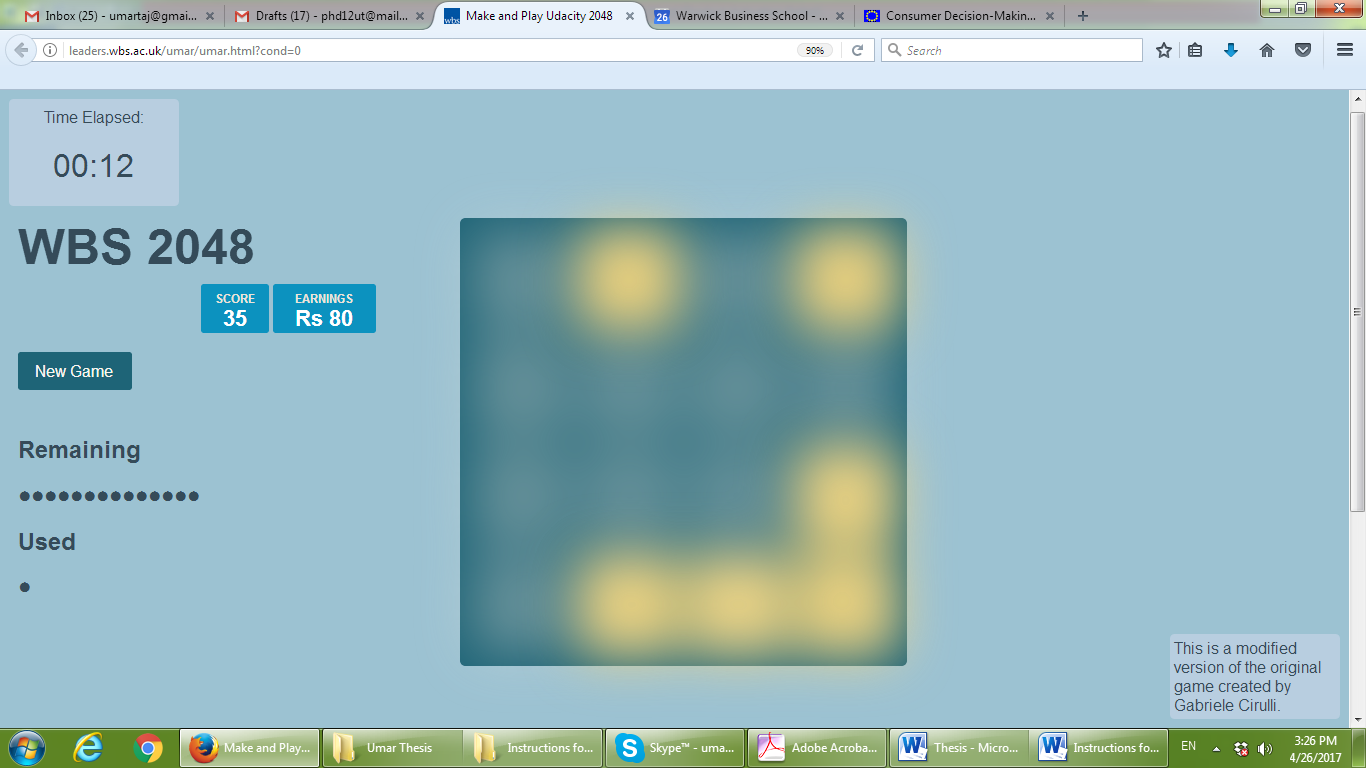 | 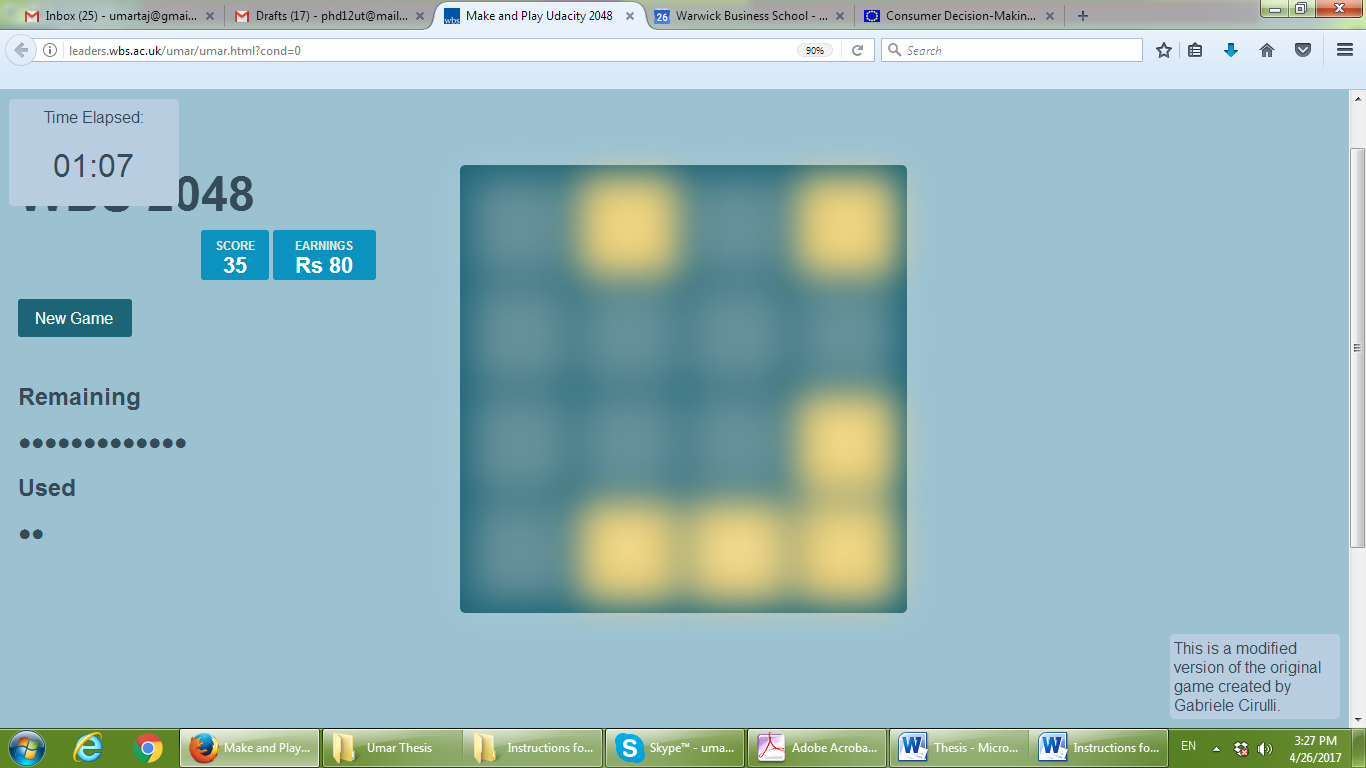 |
| --- | --- |
|  |  |
| 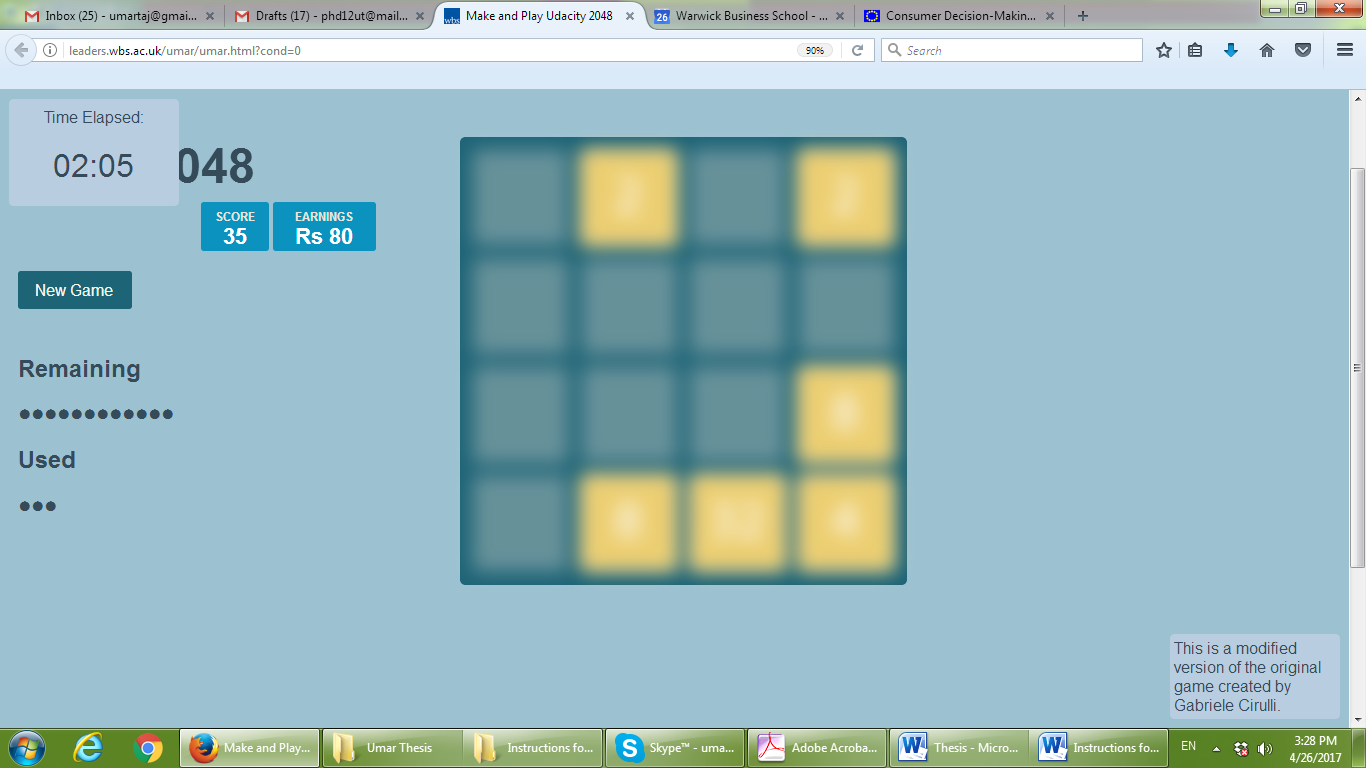 | 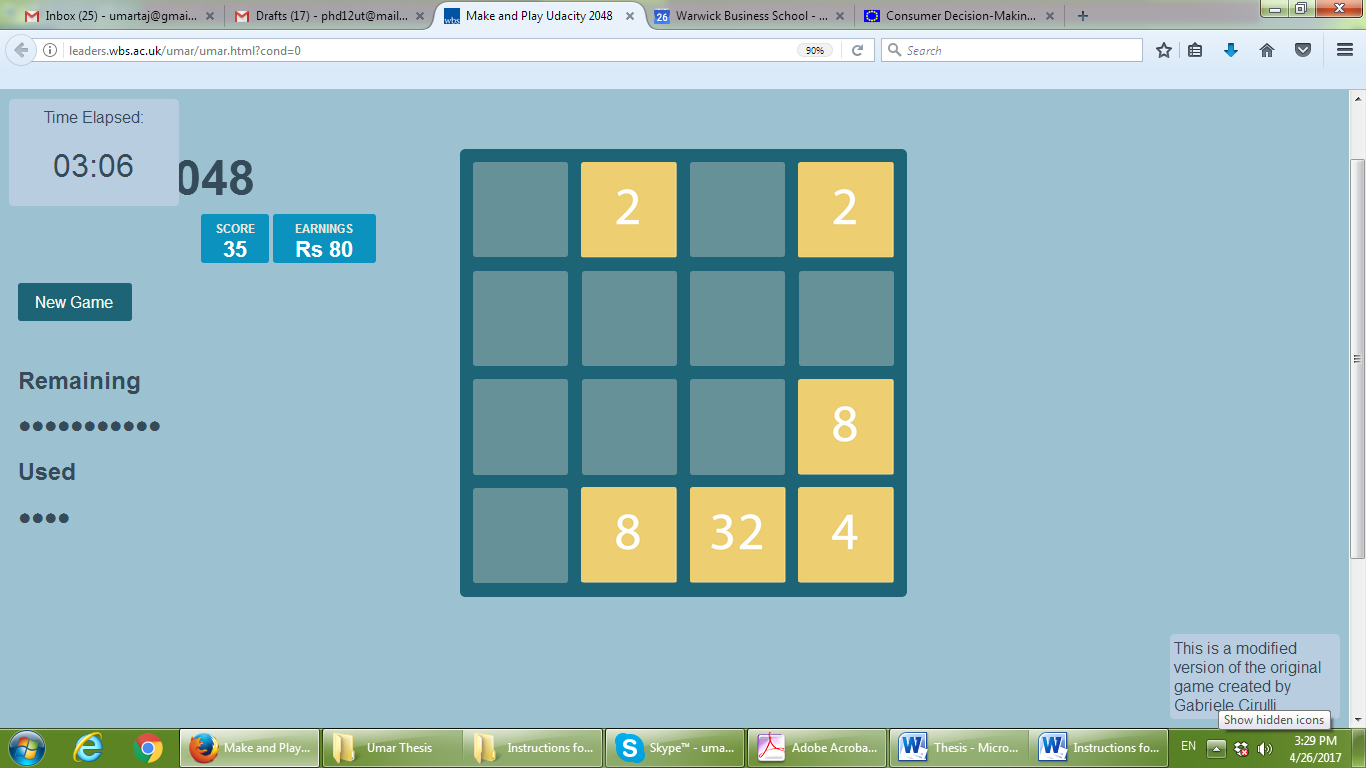 |
|  |  |
| 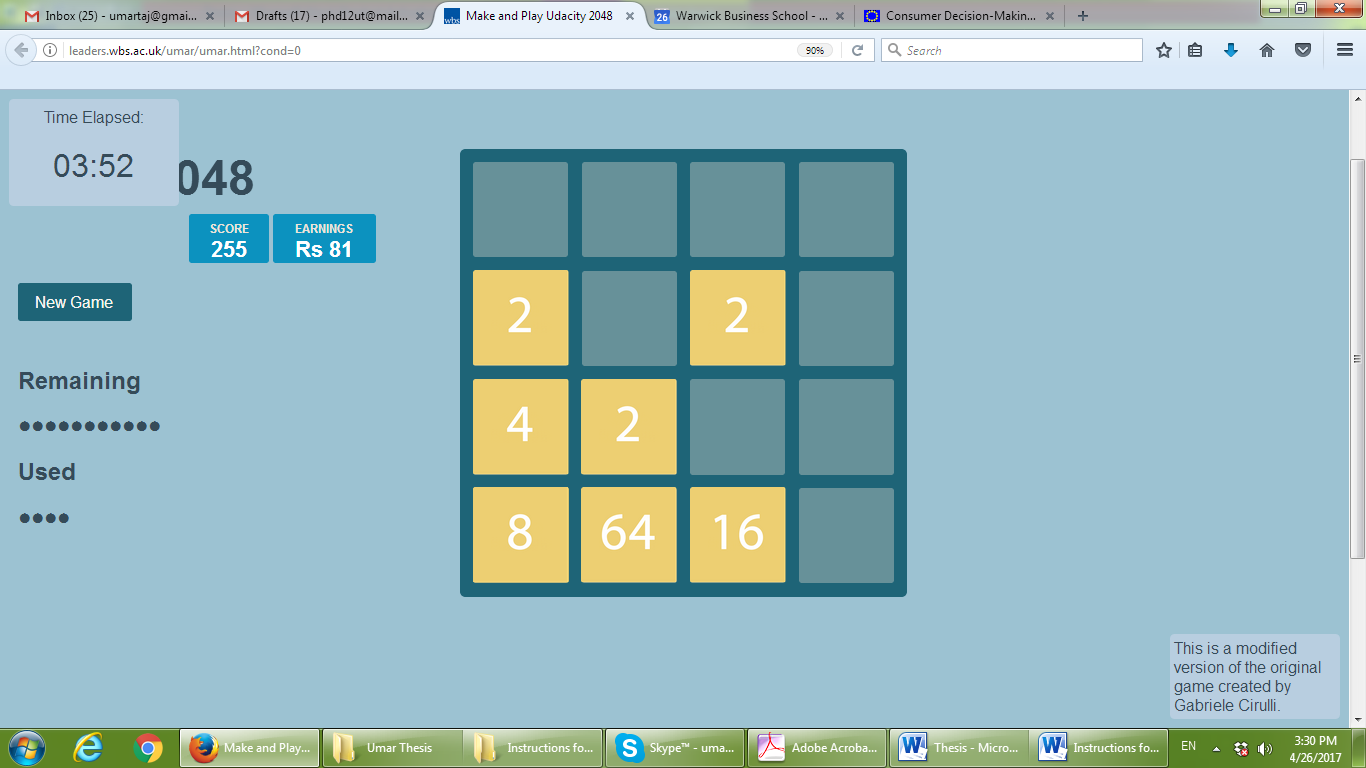 | So on . . . |
| **Figure S2.** As participants keep entering the code every minute the screen became clearer. | |

*Duration*

The total duration of the game was 14 minutes and 30 seconds, and the participants were instructed to enter the code every minute, therefore having had to enter the code 14 times in total. The 14 code entries simulated a typical seven-day antibiotic medication course where patients are prescribed to take the pill twice a day with roughly twelve-hour interval.

*Text Box to enter code*

At the bottom of the web page, a text box was placed where participants were asked to enter the code (see Figure S3). The text box was placed such that in the default web page frame participants were not able to view the text box. Participants had to scroll down each time to enter the code, and once the code was entered the web page frame would come back to the game. The reason behind placing the text box out of view of the participants was to make sure that the text box itself did not serve as a cue or reminder to enter the code. In real life setting as well, patients do not always have the pill pack in sight.

| 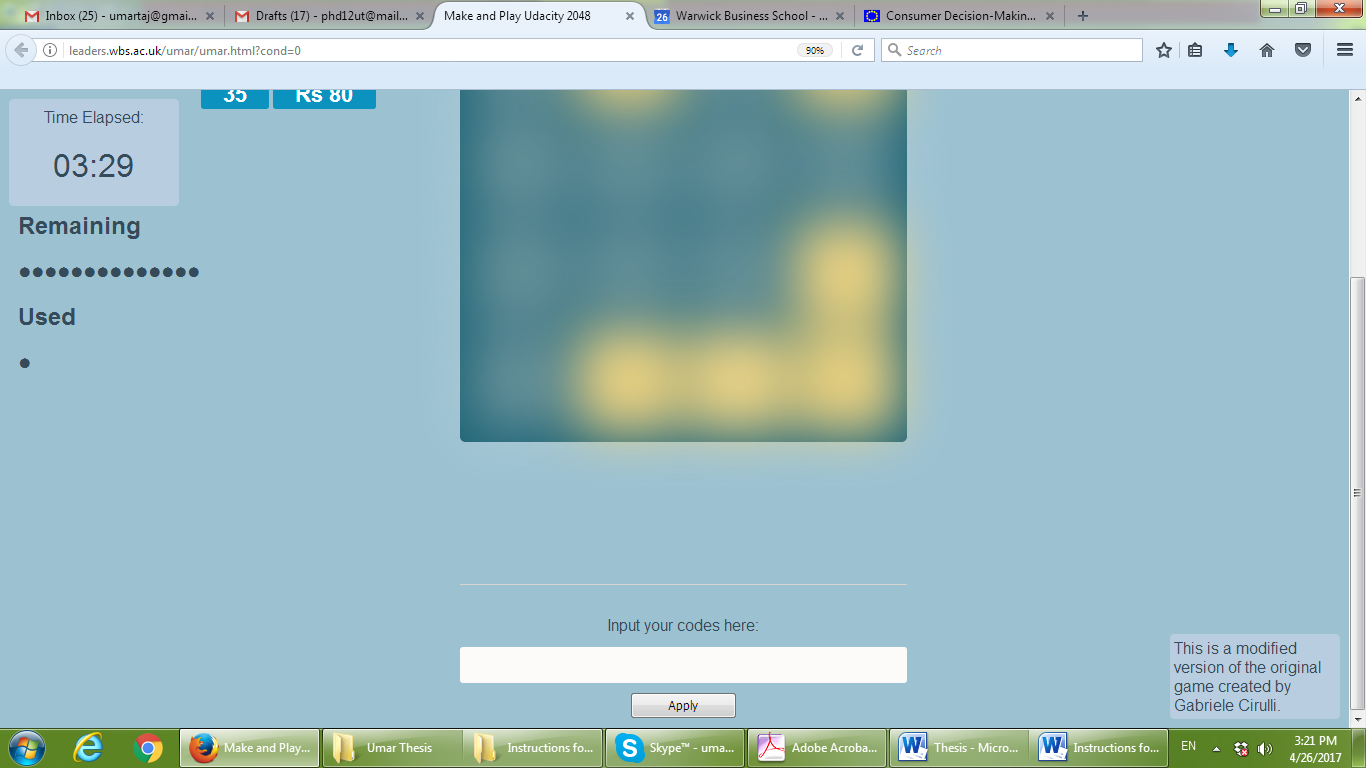 |
| --- |
| **Figure S3.** Participants had to scroll down to enter the code to clear the screen. |

*Time limit for code entries*

A code was only accepted if it was correct and entered within 15 seconds at the start of every minute. For example, for the first time if participants entered the correct code between 1 minute and 1 minute 15 seconds, then it would have reduced the blurriness. Similarly, the next time they were required to enter the code was any time between 2-minute past to 2 minute and 15 seconds past and so on.

*Scoring*

The scoring in the game was cumulative. It was based on the numbers on the tiles that merged together. So, the higher the number on the two tiles that merge together the higher was the increment in the scores.

*Code Entries*

The participants were given the code on a paper strip and the code was *sd73hp8*. The alphanumeric nature of the code made it slightly difficult for participants to type every time and the slightly longer length of the code meant that it took a little longer for them to type the code in the text box. The difficulty produced by the nature of the code and the fact that participants had to scroll down every time to enter the code provided an analogue to the cost of taking a pill such as bad taste of a pill and side effects etc. Furthermore, participants could not copy and paste the code into the text box.

When participants entered the code, a message would pop out the content of which depended on the different scenarios:

- If the code was entered correctly and within the time limit, then the message said “Correct Code (on time)”
- If the code was entered correctly but was late, then the message said “Correct Code (wrong time)”
- If an incorrect code was entered, then the message said “Incorrect Code”
- If all codes have been consumed, then the message said “No Codes Remaining”
- If the code was entered correctly twice or more within the time limit, then the message said “Correct Code (already used)”

*Pill counter*

As an analogue to the pill pack, a pill counter was displayed on the left of the screen showing how many codes have been entered and how many were remaining (see Figure S4). Participants received a total of 14 attempts to enter the code correctly, one for every minute. If participants used up all the 14 attempts before the end of the game, then their codes were ineffective even if they were entered correctly and on time subsequently.

| 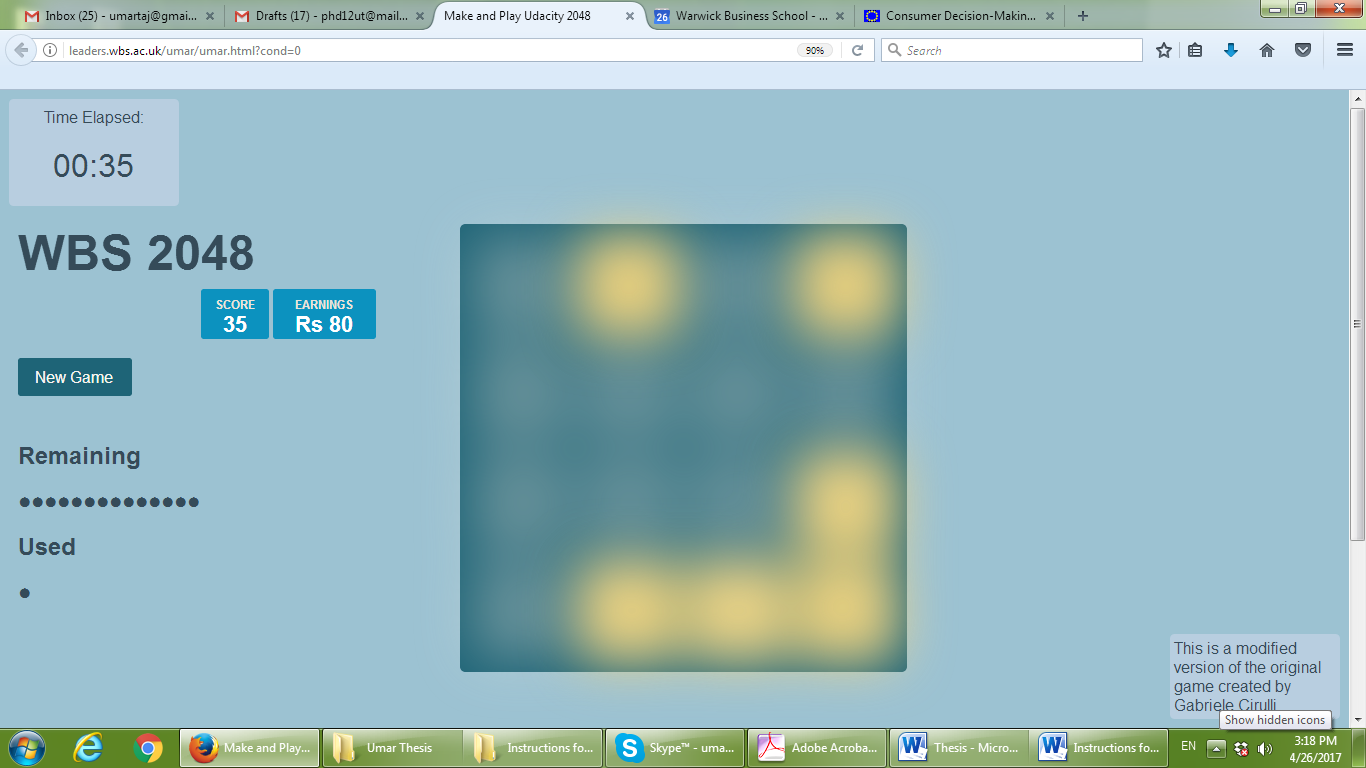 |
| --- |
| **Figure S4.** Pill counter was displayed on the left hand side of the screen. |

*Timer*

On the top left corner of the screen a timer was displayed which showed the time elapsed since the start of the game (see Figure S5). The timer was there to assist the participants in keeping track of time so that they could enter the codes on time.

| 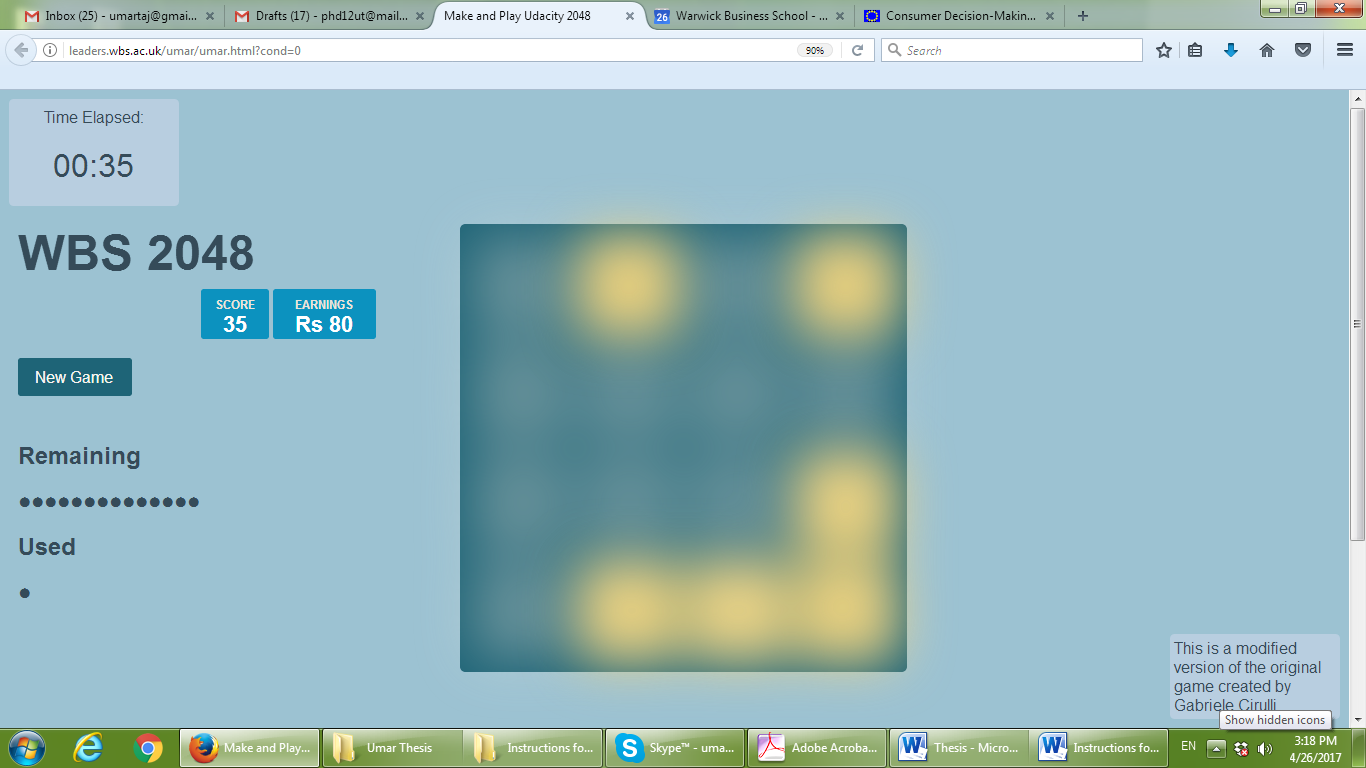 |
| --- |
| **Figure S5.** Timer was displayed on the top left hand side of the screen. |

Earnings

The show-up fee for the participants was Rupees Rs.80 (£0.50) and it was already included in the earnings; participants started the game with a score of 0 and earnings of Rs. 80. Once participants started the game, their earnings were proportional to their score.

*Game Over*

Participants were allowed to play the game as many times as possible in the duration of the experiment (which was 14 min 30 sec). Once a player had no moves left on the board, a message box popped up on the screen giving the participant an option to restart the game.

**Instruction videos**

Giving out instructions in a video format was in fact a better simulation of the real-world setting since the doctor also has verbal communication with the patient at the time of prescribing medication. Furthermore, an instruction video ensured that exactly the same content was delivered to each participant and took away any biases that we might have created whilst giving demo to the participants.

Below we describe the analogue for each of these points that were communicated to the participants of the lab experiments in the instruction video:

| **Medication instruction** | **Analogue in the instruction video** |
| --- | --- |
| Which medicine to take | Participants were given a code slip which clearly stated the code that was to be entered to help clear the screen. The instruction video referred to the code slip. |
| How often to take the medication | The instruction video clearly mentioned that the code needed to be entered every minute and two such instances were shown in the video where the code was entered and the reduction in the blurriness of the screen was observed. The window of time during which the code needed to be entered was also explained very clearly in the instruction video. |
| How long the medication should be taken | The instruction video clearly stated that the code needed to be entered 14 times in total, and that the total duration of the game was 14 minutes and 30 seconds. |
| The consequences of not completing the medication course | It was communicated to the participants in the instruction video that if they did not enter the code on time then it was likely that their screen would become blurry again. |
| The medication course must be completed and not left halfway | The participants were told in the instruction video that they must enter the code 14 times in total. |

## Piloting the 2048 experiment

We carried out some of the pilot experiments in December 2015 which is during winter in Pakistan. The lab that was set up at Gallup’s office was in a very quiet location. The experiment involved participants using the up-down-right-left arrow keys on the keyboard to play the game and the mouse attached to computer was only used to scroll down so that participants could enter the code. Since the environment was very quiet, the sound of pressing the keys on the keyboard and clicking of the computer mouse was enhanced. We were able to judge when any participant entered the code by the sound of a sudden drop of the key followed by mouse clicks. We observed during the pilot tests that these auditory changes at the time of entering the code were setting prompts for other participants to enter the code as well. During the conversation after the end of the experiment, we checked with a few participants whether the clicking sound of the mouse by the neighbouring participant gave them a cue to enter the code, however according to them that was not the case. However, just to avoid any contamination of the results the actual experiments took place during a warmer season which meant that the noise of the fan and air-conditioning effectively cancelled out the sounds from the keyboard and the mouse.

In the initial design of the experiment, the window of time that participants had to enter the code started from 10 seconds to the minute until 10 seconds after the minute. For example, for the first code entry participants could enter the code any time between 0 minutes 50 seconds until 1 minute 10 seconds and the code would work by reducing the blurriness of the screen. Some participants in the pilot tests found this window of time very confusing even though the instruction sheet clearly explained when the start and end time would be for the participants to enter the code. Upon this feedback, we changed the starting time to be exactly on the minute and then participants had 15 seconds to enter the code for it to be effective. We tested this change with a few participants in a subsequent pilot test and the matter was resolved. Hence, in the final design of the experiment, the time to enter the code started at every minute and lasted for 15 seconds.

One factor that took a bit of time to get right during the pilot tests was the analogue of the consequence of not taking the pills. In the real-world setting, the consequences of not taking the pill are issues such as relapse of the disease and increased resistance to the antibiotics, but these costs are further in the future and not very salient. We needed an analogue for the consequence of non-adherence that would not be very immediate and salient. In the initial design of the experiment, the participants were told that the number of correct on-time code entries would be recorded at the end of the experiment. We would then put a corresponding number of white paper strips in a box and then add black paper strips so that the total number of strips in the box would come to 14. Participants would then choose a strip at random and if it turns out to be a white strip then he/she would be paid the full amount earned in the game, else he/she would be paid only the show-up fee. For example, if a participant entered the correct 10 times on-time i.e. he/she took the pill only 10 times out of 14, then we would put 10 white strips and 4 black strips in a box and let the participant blindly pick a strip and pay accordingly. The results of the pilot tests with this design revealed that almost all participants were adherent. We believe that the consequence of non-adherence was very salient using the box and strips. This was more so the case because we had to demonstrate to the participants before the start of the experiment how the box and strip method would work. Even though we had this box and strip method of payment written down in the instruction sheet that we would hand out to the participants before the start of the experiment, but the participants were finding this confusing, so we started demonstrating the box and strip process. We believe that this led to the consequence of non-adherence becoming very salient and hence not the best of analogues for our experimental game modelling. Since the adherence rates that we were getting in our pilots of the control experiment were very high, they were not really comparable to the adherence rates observed in real world.

We then moved to the other extreme and carried out a few pilot tests with no consequence of non-adherence (all else remained the same). The results showed that there was much more non-adherence in these pilot test which was expected as there was no consequence of not entering the code. However, there was a serious criticism to this approach as there was no analogue of consequence and only the people in the incentive treatment group would have an incentive to take the medication and thereby enter the code.

The final design of the experiment incorporated the consequence of non-adherence in the following way: When participants started the game, the blurriness was set at 100% and each time they entered the correct code the blurriness was reduced by 25%. If they failed to enter the code, there was a 2% chance of a “relapse” where the screen became blurry by 25%. The probability of a “relapse” doubled each time the code was not entered. This mechanism of “relapse” activated in the experiment after the screen became clear for the first time. If a “relapse” had occurred, the participants were able to clear the screen again by subsequently entering a correct code on time.

### 2.12 Game play

Since most of the people participating in our lab experiments were not familiar with the game 2048, we first showed them how the original game is played and gave them practice time. There is a very quick learning curve for 2048 and after about 10 minutes of play every participant was quite comfortable with the game. In our initial pilot tests, we showed participants how to play the original 2048 game, gave them practice time, handed out the instruction sheet and then asked them to start playing the experimental game. However, since there was a considerable difference between the original 2048 game and the modified version (which included the blurriness aspect and code entry) many participants got confused and did not understand the code entering process. Upon this feedback, we included a demonstration of how to play in the actual experiment as part of the participant’s orientation. So, for each of the control or treatment conditions, we would show them on a multimedia how to play in that particular control or treatment condition and explained the features that were different from the original game such as timer, pill counter, blurriness and entering the code. In essence, we showed a demo of the instruction sheet that was provided to the participant. This short demo of how to play in the actual experiment improved the modelling of the real-world scenario. People are quite used to taking an antibiotic medication course and it is not a novel exercise. Most of the participants in the experiments were playing the original 2048 game for the first time and none of them had played the modified version of the game; the experimental game was completely new to them. As a result, introducing the practice session to play the actual 2048 game and giving a demo of what to expect and how to play the modified version in our experiments made a stronger case for an analogue to the real-world setting.

In order to ensure that exactly the same demo was given every time before the start of the experiment, we recorded a video of the game screen with the researcher voice over. This video was used in all the actual experiments that were run.

### 2.13 Procedure

The first screening criterion to recruit participants for our lab experiments was for them to be computer literate. For the initial pilot tests, we prepared the instruction sheet in English assuming that if participants are computer literate then they would be able to read and understand English as well. We observed in the first few pilot tests that all participants were able to read the English instruction sheet but some of them did not really understand the instructions. We realized this from the way they played in the experimental game and also from having a focus group conversation with them after. In order to avoid any misunderstanding of the instruction sheet, we prepared an Urdu version of the instruction sheet as well and at the start of the pilot tests, offered participants the choice between Urdu and English instruction sheets. We realized that when we offered the choice between Urdu and English instruction sheets, all the participants opted for the English instruction sheet even though we knew that some of the participants might not be able to completely understand the instructions in English. This behaviour is likely to derive from the perception of English as a status symbol in Pakistani society. Oftentimes, knowledge of the English language is used as a measure of an elite and socially acceptable education. This is reflected by English being the official language of Pakistan (even though the national language is Urdu). We observed that some of the participants in our pilot tests chose the English instruction sheets (even though some of them did not completely understand the English instructions) to signal to the researcher and the rest of the group that they are proficient in the language.

We wanted to make sure that all participants completely understood the instructions and the game play of our experiment while avoiding the English/Urdu instruction sheet problem. We therefore decided to develop an instruction video in Urdu explaining all the content that was written in the instruction sheet. The video also provided the opportunity to include the demo of the actual experiment. Furthermore, in the real-world setting a verbal communication takes place between the GP and the patient at the time of antibiotic prescription (in Pakistan, the language used in Urdu). Therefore, using an instruction video made a stronger case for an analogue to the real-world setting (see Figure 12).

In one of the pilot tests the participants knew each other, and we observed after the end of the experiment they were comparing their scores and also how many times they entered the code. One of the participants commented that he stopped entering the code because the screen was clear. We interviewed another participant after the experiment finished and she stated that she remembered that if she did not enter the code the screen would become blurry again (this demonstrated that she knew the consequence of non-adherence) but then she missed the code entry one time and nothing happened so she relaxed thereafter. One other common theme that emerged from interviewing the participants was that they simply forgot to enter the code because they were engaged in the game. We acknowledge of course that these qualitative statements are not enough to justify the validity of the modelling experiment (in terms of its simulation of patient’s behaviour) but it was a very positive sign to see that the experiment was generating the same behaviour and feeling as were noted among the patients.

Gallup Pakistan recruited the participants based on the demographic profile that we shared with them. To many of the participants, Gallup provided a transport facility to make it easy for them to participate in the study.

The arrangement of the room is described below.

Each desk had:

- A laptop;
- Keyboard;
- Mouse;
- Internet connection;
- Google Chrome on the computer system and
- Headphones.

On every laptop an incognito window was opened in Google Chrome. In this window, three tabs were opened:

- A tab with the original 2048 game: <https://gabrielecirulli.github.io/2048/>
- A tab with the instruction video
- A tab with the study game: <http://leaders.wbs.ac.uk/umar/index.html?cond=X>; where X was replaced with the condition number that the participant was playing. So, if it was Condition 0 then X was replaced with 0 and so on…

There was a separating mechanism between each desk so that the participants were unable to see each other’s screens.

PARTICIPANT INFORMATION LEAFLET

| Study Title: | Decision Making Study | |
| --- | --- | --- |
| Investigator(s): | *ANNONYMISED* | |
| Introduction  You are invited to take part in a research study. Before you decide, you need to understand why the research is being done and what it would involve for you. Please take the time to read the following information carefully. Talk to others about the study if you wish.  (Part 1 tells you the purpose of the study and what will happen to you if you take part. Part 2 gives you more detailed information about the conduct of the study)  Please ask us if there is anything that is not clear or if you would like more information. Take time to decide whether or not you wish to take part. | |  |

PART 1

| What is the study about?  We would like to understand how people make decisions in a game setting. |
| --- |
| Do I have to take part?  It is entirely up to you. We will describe the study and go through this information sheet, which we will give you to keep. If you choose to participate, we will ask you to sign a consent form to confirm that you have agreed to take part (if part of this study is an online or postal questionnaire/survey, by returning a completed questionnaire/survey, you are giving your consent for the information that you have supplied to be used in this study and formal signed consent will not be collected where postal or online questionnaires/surveys are concerned). You will be free to withdraw at any time, without giving a reason and this will not affect you or your circumstances in any way. |
| What will happen to me if I take part?  You will be playing a modified version of a game called 2048. In this game you combine numbered tiles (using the up-down-right-left arrow keys) in an attempt to reach 2048. |
| What are the possible disadvantages, side effects, risks, and/or discomforts of taking part in this study?  There are no physical risks beyond normal computer use |
| What are the possible benefits of taking part in this study?  We are excited about what we can learn from the data collected in this study and we will use the information to improve our understanding of human decision making. The research also has applications for use in public policy and industry. |
| Expenses and payments  You will receive Rs. 80 show-up fees and can earn up to a maximum of Rs. 500 based on how well you perform in the game. |
| What will happen when the study ends?  We will be analysing the data collected to understand how people in general make decisions. |
| Will my taking part be kept confidential?  Yes. We will follow strict ethical and legal practice and all information about you will be handled in confidence. Further details are included in Part 2. |
| What if there is a problem?  Any complaint about the way you have been dealt with during the study or any possible harm that you might suffer will be addressed. Detailed information is given in Part 2. |

This concludes Part 1.

If the information in Part 1 has interested you and you are considering participation, please read the additional information in Part 2 before making any decision.

PART 2

| Who is organising and funding the study?  The study is being organised and funded by researchers at *ANNONYMISED*. |
| --- |
| What will happen if I don’t want to carry on being part of the study?  Participation in this study is entirely voluntary. Refusal to participate will not affect you in any way. If you decide to take part in the study, you will need to sign a consent form, which states that you have given your consent to participate.  If you agree to participate, you may nevertheless withdraw from the study at any time without affecting you in any way.  You have the right to withdraw from the study completely and decline any further contact by study staff after you withdraw. |
| What if there is a problem?  This study is covered by the University of *ANNONYMISED*’s insurance and indemnity cover. If you have an issue, please contact the Chief Investigator of the study:  *ANONYMISED* |
| Who should I contact if I wish to make a complaint?  Should anyone have any complaints relating to a study conducted at the University or by *ANNONYMISED* University's employees or students, the complainant should be advised to contact the Director of Delivery Assurance, details as below:  Director of Delivery Assurance Registrar's Office  *ANNONYMISED* |
| Will my taking part be kept confidential?  Your performance in this study is completely confidential and will be stored anonymously. Your identity will not be shared with anyone else. Data will be kept securely after the study. The form signed at the end of the study is just for the accounts department to keep track of the University's funds. |
| What will happen to the results of the study?  The data that will be collected in this study may be used to publish in an academic journal. |

| Who has reviewed the study?  This study has been reviewed and given favourable opinion by the *ANNONYMISED*’s Humanities and Social Science Research Ethics Committee (HSSREC): |
| --- |
| What if I want more information about the study?  If you have any questions about any aspect of the study, or your participation in it, not answered by this participant information leaflet, please contact:  *ANNONYMISED* |

Thank you for taking the time to read this participant information leaflet.

HUMANITIES AND SOCIAL SCIENCE ETHICS COMMITTEE CONSENT FORM

Study Number:

Title of Project: Decision Making Study

Name of Researcher(s): *ANNONYMISED*

Please initial all boxes

I confirm that I have read and understand the information sheet dated [DATE] for the above study. I have had the opportunity to consider the information, ask questions and have had these answered satisfactorily.

I understand that my participation is voluntary and that I am free to withdraw at any time without giving any reason, without my medical, social care, education, or legal rights* (**delete as appropriate*) being affected.

I agree to take part in the above study.

Name of Participant Date Signature

Name of Person Date Signature

taking consent
